# Performance Evaluation of Target Detection with a Near-Space Vehicle-Borne Radar in Blackout Condition

**DOI:** 10.3390/s16010064

**Published:** 2016-01-06

**Authors:** Yanpeng Li, Xiang Li, Hongqiang Wang, Bin Deng, Yuliang Qin

**Affiliations:** School of Electrical Science and Engineering, National University of Defense Technology, 137 Yanwachi Street, Changsha 410073, China; lixiang01@vip.sina.com (X.L.); oliverwhq@vip.tom.com (H.W.); dengbin@nudt.edu.cn (B.D.); nudtqyliang@163.com (Y.Q.)

**Keywords:** blackout, near-space vehicle, near-space vehicle-borne radar, performance evaluation, radar, target detection

## Abstract

Radar is a very important sensor in surveillance applications. Near-space vehicle-borne radar (NSVBR) is a novel installation of a radar system, which offers many benefits, like being highly suited to the remote sensing of extremely large areas, having a rapidly deployable capability and having low vulnerability to electronic countermeasures. Unfortunately, a target detection challenge arises because of complicated scenarios, such as nuclear blackout, rain attenuation, *etc*. In these cases, extra care is needed to evaluate the detection performance in blackout situations, since this a classical problem along with the application of an NSVBR. However, the existing evaluation measures are the probability of detection and the receiver operating curve (ROC), which cannot offer detailed information in such a complicated application. This work focuses on such requirements. We first investigate the effect of blackout on an electromagnetic wave. Performance evaluation indexes are then built: three evaluation indexes on the detection capability and two evaluation indexes on the robustness of the detection process. Simulation results show that the proposed measure will offer information on the detailed performance of detection. These measures are therefore very useful in detecting the target of interest in a remote sensing system and are helpful for both the NSVBR designers and users.

## 1. Introduction

The fundamental functions of a radar are searching/detecting, tracking and imaging [1]. Therefore, numerous remote sensing applications can be satisfied by the utilization of a radar [1]. In the last 60 years, radars can be classified into surface-based (ground-based and sea-based, including vehicle-borne and ship-based) radar, airborne radar and space-based radar, according to the installation or platform [1,2]. As a supplemental installation, near-space vehicle-borne radars have ignited great interest for surveillance applications in recent years [3,4,5,6]. For surveillance purposes, a near-space vehicle-borne radar has many advantages to be addressed:

- Compared to a surface-based radar, the near-space vehicle-borne radar has wide coverage, as it is capable of the surveillance of extremely large areas [5,7].
- Compared to current airborne and spaceborne radars, the near-space vehicle-borne radar (NSVBR) obtains higher revisiting frequency, higher resolution and more robust survivability [3,8].
- Depending on the carrier’s high maneuvering characteristic, NSVBR has low vulnerability to electronic countermeasures [4].
- For the application in complicated missions, NSVBR’s rapidly deployable capability enables it the best achievable detection for long-range targets [9].

**Figure 1:**
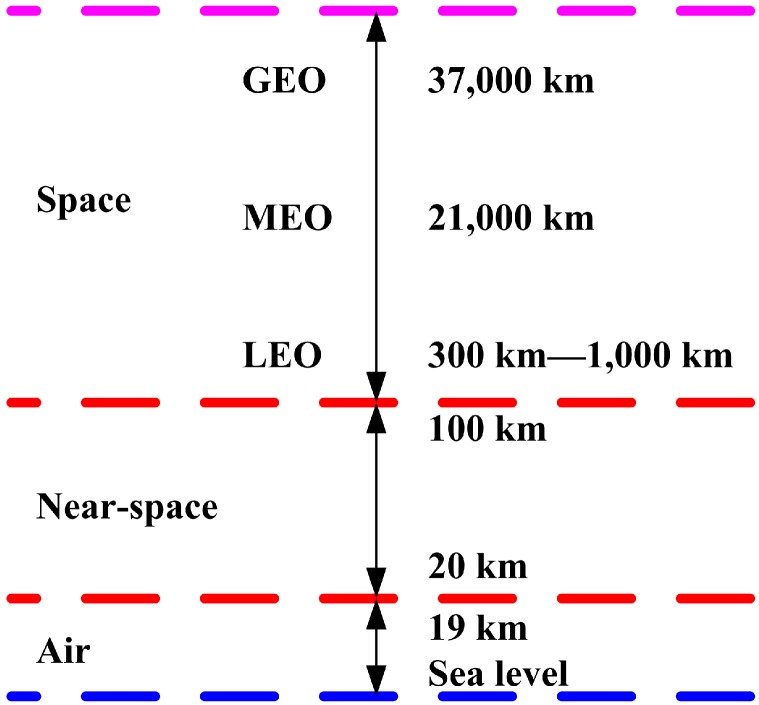
Definition of the near space. GEO, geosynchronous orbit; MEO, middle Earth orbit; LEO, low Earth orbit.

Figure 1 shows the definition of the near-space where the above-mentioned advantages of the near-space can be addressed [5]. Herein, GEO is the geosynchronous orbit; MEO is the middle Earth orbit; and LEO is the low Earth orbit.

Given the above listed advantages, however, at times, practical considerations—designation issues, cost, *etc*.—affect the implement of an NSVBR. The main technology difficulties are: the “nuclear blackout” (also known as radio blackout, ionization blackout or sometimes reentry blackout; may be called “blackout” for short) effect originating from high-altitude nuclear bursts and high-speed flying [2], the rain attenuation when sensing low-altitude flying targets and/or ground targets [5,7], the multi-path interference when applied at low grazing angles [8], *etc*. In these problems, the blackout effect is ubiquitous in almost all applications of an NSVBR, and field tests cannot be performed with ground-based equipments. Therefore, the analysis of the detection capability (particularly in blackout condition) for an NSVBR is of great importance for the implementation of such a system.

Research on NSVBR is in its infancy, and most of the relevant studies discuss the overall system designation [5]. It is strongly emphasized that, for remote sensing, the NSVBR fills the gap between ground-based radar, space-borne radar and airborne radar [5,9,10,11,12].

A widely-discussed topic is near-space vehicle-borne synthetic aperture radar (SAR) [7,8,13,14]. To provide a promising solution for remote sensing applications, a digital beamforming-based near-space wide swath imaging technique is developed, where multiple beams in the azimuth are used to suppress the possible azimuth ambiguities for near-space high-resolution and wide-swath SAR imaging [13]. For regional remote sensing surveillance, a distributed passive radar sensor network with near-space vehicle-borne receivers is studied [9,12]. Because there is a large speed difference between the transmit and the receive platforms, a multi-beamforming and scan-on-receive combined approach is presented to extend the limited imaging coverage [9]. A new concept for tsunami detection is proposed with the application of an NSVBR [11], whose advantage point is that it is conceived of to protect the population from local tsunamis.

There are some works on the analysis of the effects of blackout on the propagation degradation of electromagnetic (EM) waves [2,15,16,17]. It is figured out that the blackout attenuation has a relationship with the frequency of the EM wave, the frequency of the plasma and the collision frequency of the plasma [15]. For a navigation system whose signal passes through the nuclear blackout environment, some researchers try to investigate techniques that may overcome this phenomenon [16,17].

In the performance evaluation of target detection with a radar installed on other platforms, the widely-employed evaluation index is the probability of detection and another approach called the receiver operating curve [1,2]. Though they can give overall results of detecting a target for an NSVBR, however, no in-depth information is given related to the effects of blackout on the propagation degradation of EM waves. In addition to these works, there is target location and accuracy analysis in near space bistatic radar [18]. The precision of position estimation is calculated [18].

Based on the materials described above, it can be seen that NSVBR is a recently-proposed designation, and the available literature is limited; the close-related research on the performance analysis of the detection capability are even limited. The existing two performance evaluation approaches for target detection cannot offer detailed performance information in this complicated scenario. With the knowledge of performance evaluation for automatic target recognition systems [19] and previous work on radar signal processing [20,21], we developed some evaluation indexes that may fulfill the performance evaluation demand of target detection with an NSVBR. The objectiveness in each section and the data are organized as follows:

- We first review the background of an NSVBR and the existing methods in the performance evaluation of target detection with a radar. The research objectiveness is then explained. These are the main contents in Section 1.
- Section 2 details the target detection problem, the effect of the blackout on radar target detection and the performance evaluation of radar target detection in an NSVBR, serving as the background of this work.
- The majority of our work, Section 3, presents the performance evaluation indexes on the detection capability of an NSVBR. In a blackout situation, both the detection ability and the robustness of the detection process can be considered by these indexes.
- In order to know the performance of this newly-developed methodology, we performed some experiments. Section 4 presented the simulation results and the analysis of them. Based on the experimental results, there is a detailed discussion in Section 5 between our novel approaches and the existing work.
- Section 6 highlights the contribution of this paper and the future work.

## 2. Problem Formulation and the Background

### 2.1. Target Detection with a Near-Space Vehicle-Borne Radar

Like the detection course in other radar systems, target detection with an NSVBR is a decision problem that is realized by establishing a threshold signal level (voltage) on the basis of the current interference voltage [1]. Then, the detector will make a decision on the presence of a target by comparing the signal level with that threshold. When the signal level exceeds the threshold, the presence of a target is declared; while if the signal does not exceed the threshold, then no target is declared [1].

Compared to a radar system installed on other platforms, an NSVBR experiences some challenges in correctly detecting a target [5,12]. Because there are more issues that should be described than can be presented here, some important facts will be mentioned. First, the blackout makes the scenario complicated: the echo of the target is attenuated; the signal-to-noise ratio (SNR) is then lowered; the blackout may even introduce additional phase variation, which affects the coherent integration. Second, the rain attenuation also brings disadvantage to the detection course by lowering the SNR. Third, if there is severe multi-path interference, a false alarm rate may arise and the detection performance will hence degrade.

### 2.2. Effect of the Blackout on Radar Target Detection Performance

The so-called blackout is caused by an envelope of ionized air (that is created by the heat from the friction of the vehicle against the atmosphere) around the flying vehicle [2,15,16,17]. The blackout may result in reflectance and/or a high degrees of attenuation on radar operation, which greatly prevent the detection of targets of interest [2,15]. Experts make their efforts on modeling this influence, which may help to quantify the problem. For a given scenario, the attenuation model of blackout on radar echoes is:

(1) $$\alpha =\frac{8.68\omega }{c\sqrt{2}}{\left[\sqrt{{\left(1-\frac{\omega_{p}^{2}}{\omega^{2}+\nu_{1}^{2}}\right)}^{2}+{\left(\frac{\omega_{p}^{2}}{\omega^{2}+\nu_{1}^{2}}\cdot \frac{\nu_{1}}{\omega }\right)}^{2}}-\left(1-\frac{\omega_{p}^{2}}{\omega^{2}+\nu_{1}^{2}}\right)\right]}^{\frac{1}{2}}$$

where *α* is the constant attenuation factor whose unity (dimension) is dB/m [15]. *ω* is the frequency (of the transmitted electromagnetic wave) in radians per second (rad/s). $\omega_{p}$ is the frequency of the plasma (rad/s). *c* is the speed of light in a vacuum (m/s). $\nu_{1}$ is the collision frequency of the plasma (1/s). The total attenuation (of the blackout effect) is the integration along the propagation path through the plasma. We found that, to be reasonable with dimensional analysis in calculation, $\nu_{1}$ should be replaced by $\nu =2\pi \nu_{1}$. Therefore, ωp2ωp2ω2+v2ω2+v2 and vvωω are dimensionless values, which facilitates the mathematical derivations.

(2) $$\alpha =\frac{8.68\omega }{2\pi c\sqrt{2}}{\left[\sqrt{{\left(1-\frac{\omega_{p}^{2}}{\omega^{2}+\nu^{2}}\right)}^{2}+{\left(\frac{\omega_{p}^{2}}{\omega^{2}+\nu^{2}}\cdot \frac{\nu }{\omega }\right)}^{2}}-\left(1-\frac{\omega_{p}^{2}}{\omega^{2}+\nu^{2}}\right)\right]}^{\frac{1}{2}}$$

The integration along the propagation path through the plasma of *α* will be the value of attenuation. This model will be utilized in the coming simulation.

### 2.3. Performance Evaluation of Radar Target Detection

Compared to the performance evaluation measures of radar target tracking and radar target recognition [22,23], the methods of the performance evaluation of radar target detection are limited. There are two widely-used measures: the probability of detection ($P_{D}$) and the ROC method [1]. Similar to the index of $P_{D}$, the probability of false alarm, $P_{FA}$, can be resolved [1].

There are two ways to resolve the probability of detection and the probability of false alarm [24,25]. In the context of radar signal processing, the probability of detection is determined by the integration (of the signal-plus-noise’s probability density function) from the threshold voltage to positive infinity [1]:

(3) $$P_{D}=\int_{v_{t}}^{\infty }p_{s+i}\left(v\right)dv$$

where $v_{t}$ is the threshold voltage and $p_{s+i}\left(v\right)$ is the signal-plus-noise’s probability density function. Similarly, the probability of a false alarm is found from [1]:

(4) $$P_{FA}=\int_{v_{t}}^{\infty }p_{i}\left(v\right)dv$$

where $p_{i}\left(v\right)$ is the noise probability density function.

In the context of probability and statistical inference, however, $P_{D}$ is the ratio of the number of the correctly-detected targets to the number of the targets in the region of interest. $P_{FA}$ is thus the ratio of the number of the incorrectly-detected targets to the number of the targets in the region of interest. These two results are in fact the posterior probability. We will not introduce this method in our work.

In order to describe the trade-off between detection and false alarm probabilities for a given SNR, the ROC method is developed [1]. Classically, for a nonfluctuating target, ROC presents a set of curves of the SNR required to achieve a given $P_{D}$, with $P_{FA}$ as a parameter.

## 3. The Novel Performance Evaluation Indexes on Detection Capability

Because the near-space vehicle-borne radar is a new application of radar systems and it usually works in more complicated scenarios, the performance evaluation indexes on detection ability should be different from the conventional metrics, which typically evaluate with the probability of detection. The novel evaluation indexes should also take into account the robustness of the detection course. Therefore, novel performance evaluation indexes on detection capability can be developed with statistical methodologies.

### 3.1. Evaluation Index on Detection Probability

The widely-used evaluation index, probability of detection, is relatively very easy to calculate. However, two important parameters have been neglected. In particular, the transmitted power of the radar greatly influences the strength of the signal coming into the radar receiver from the target of interest, and hence, the transmitted power affects the overall SNR. Therefore, the hypothesis test in the detecting course is affected. Another interesting aspect is the radar cross-section (RCS) of the target of interest. This parameter has a similar impact on the detecting course. In view of the above-mentioned facts, we developed a novel metric, the ratio of the probability of detection, to the product of the transmitted power ($P_{t}$) and the target’s RCS (*σ*),

(5) $$\eta_{\mathrm{detection}}\overset{\Delta }{=}\frac{P_{D}}{P_{t}\sigma }$$

This novel evaluation index, $\eta_{\mathrm{detection}}$, is referred to as the “normalized probability of detection (NPD)” in our work. It can be seen that all of the probability of detection, the transmitted power and the target’s RCS are considered in this index in a practical way.

To apply this index in the evaluation work, three metrics are calculated to characterize the performance in different technical specialties: NPD in a single detection procession (NPD-S), averaged NPD with respect to the entire operation (mission) (NPD-M) and averaged NPD with regard to the altitude (NPD-A).

Suppose that there is a total of *N* times detection procession in an operation (mission); NPD-S is defined to be:

(6) $$\eta_{\mathrm{detection}}\left(k\right)\overset{\Delta }{=}\frac{P_{D}\left(k\right)}{P_{t}\left(k\right)\sigma \left(k\right)},k=1,\;2,\;\ldots ,\;N$$

where $P_{D}\left(k\right)$ is the probability of detection at time *k* and $P_{t}\left(k\right)$ is the corresponding transmitted power (sometimes an uniform illumination is utilized). σ(k) is the target’s RCS at time *k*. NPD-S is used to show the step-by-step detection ability.

With regard to its physical implication, NPD-M is arrived at by:

(7) $${\bar{\eta }}_{\mathrm{detection}}\left|{}_{\mathrm{operation}}\right.\overset{\Delta }{=}\frac{1}{N}\sum_{k=1}^{N}\eta_{\mathrm{detection}}\left(k\right)$$

To resolve NPD-A, we first divide the near-space vehicle’s operating altitude (from 10 km to 110 km above sea level) into 100 shares. For the *i*-th share, suppose that there is a total of $m_{i}$ times detection procession throughout the operation (mission); NPD-A thus reads:

(8) $${\bar{\eta }}_{\mathrm{detection}}\left|{}_{\mathrm{altitude}}\left(i\right)\right.\overset{\Delta }{=}\frac{1}{m_{i}}\sum_{k=1}^{m_{i}}\eta_{\mathrm{detection}}\left(k\right),\;i=1,\;2,\;\ldots ,\;100$$

Because of the high maneuvering characteristic of the near-space vehicle, the radar may experience various altitudes (which means different parameters in Equation (2)) in surveillance. As a result, acknowledging the performance in various altitudes is of great importance in this work.

### 3.2. Evaluation Index on the Robustness of the Detection Process

For a near-space vehicle-borne radar, it ideally should be able to deal with widely-varying environment (blackout, rain, multi-path, *etc*.) and target characteristics (size, range, aspect, heading, bearing, altitude, speed, maneuvering, *etc*.). Knowing the robustness of the detection process is therefore the most fundamental task in evaluating the performance of detection. We will model the robustness of the detection process with two indexes: the robustness of NPD as a function of time (RNPD-T) and the robustness of NPD as a function of altitude (RNPD-A).

RNPD-T ($\varsigma \left|{}_{\mathrm{time}}\right.$) takes the form:

(9) $$\varsigma \left|{}_{\mathrm{time}}\right.=1-\frac{\sqrt{\sum_{k=1}^{N}{\left(\eta_{\mathrm{detection}}\left(k\right)-{\bar{\eta }}_{\mathrm{detection}}\left|{}_{\mathrm{operation}}\right.\right)}^{2}}}{N{\bar{\eta }}_{\mathrm{detection}}\left|{}_{\mathrm{operation}}\right.}$$

RNPD-T provides information on the variation of detecting performance regarding time.

RNPD-A ($\varsigma \left|{}_{\mathrm{altitude}}\right.$) is given by:

(10) $$\varsigma \left|{}_{\mathrm{altitude}}\right.=1-\frac{\sqrt{\sum_{i=1}^{100}{\left({\bar{\eta }}_{\mathrm{detection}}\left|{}_{\mathrm{altitude}}\left(i\right)\right.-{\bar{\eta }}_{\mathrm{detection}}\left|{}_{\mathrm{operation}}\right.\right)}^{2}}}{100{\bar{\eta }}_{\mathrm{detection}}\left|{}_{\mathrm{operation}}\right.}$$

The variation of detecting performance regarding altitude is effectively presented by RNPD-A.

### 3.3. Summary of the Proposed Method

**Figure 2:**
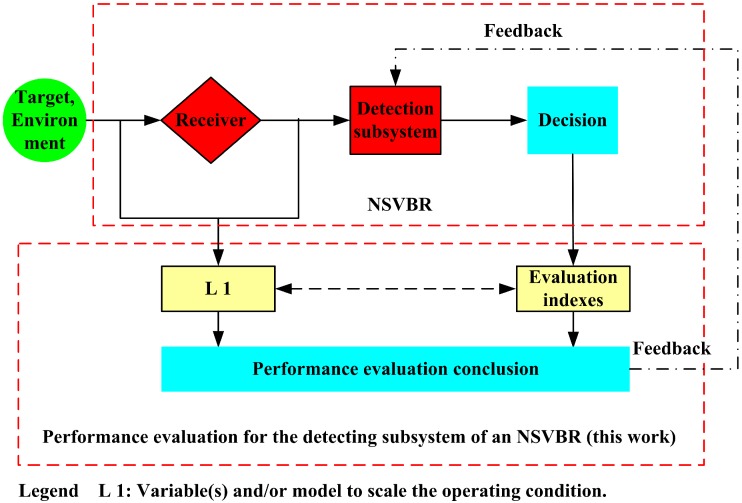
The block diagram of this work and the relationship between a near-space vehicle-borne radar (NSVBR) and this work.

We now can describe the detecting performance of an NSVBR in blackout condition with the theoretical part of this work; the idea and the main contribution is presented in Figure 2. It can be known that the operating condition is scaled by $P_{t}$ and *σ*. The evaluation indexes are NPD, NPD-S, NPD-M, NPD-A, RNPD-T and RNPD-A. An objective conclusion may be drawn with these facilities.

## 4. Results of Validation Experiments

To know the applicability of the proposed performance evaluation methodologies, experiments are performed.

### 4.1. Experimental Setup

**Figure 3:**
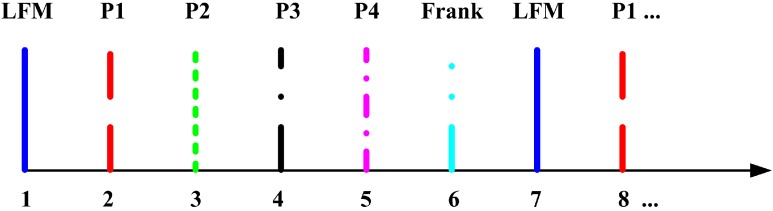
The “time-division multiple waveform” work mode.

There are three scenarios taken into account: an X-band NSVBR (Scenario 1; 10.00–10.03 GHz), a Ku-band NSVBR (Scenario 2; 15.00–15.03 GHz) and a Ka-band NSVBR (Scenario 3; 30.00–30.03 GHz). For each scenario, there are 5000 detection processions. In these, the waveforms of the radar are linear frequency modulation (LFM), P1 code, P2 code, P3 code, P4 code and Frank code. The radar works in a “time-division multiple waveform” mode, as is shown in Figure 3. For each waveform, the pulse repetition frequency (PRF) is 1 kHz. Therefore, the illumination PRF (regardless of waveform) of the radar system is 6 kHz. For each mentioned carrier frequency, the average transmitted power is identically 0.5 kw, while the peak transmitted power ($P_{t}$) is identically 100 kw.

The RCS of the target is 0.03 dBsm–0.1 dBsm (a dim target), as a function of aspect angle. To facilitate the experiment, the RCS series are simulated according to the Swerling 1 distribution [1]. Because of the relatively low SNR of the echo from this dim target, a coherent integration is implemented with 10 echoes. The gain of integration for the improvement in SNR is approximately 7 dB. To avoid any possible confusion in this paper, the value of SNR is the SNR of the received signal, unless otherwise stated.

SNR is resolved according to Equations (8) and (10) to Equation (11) in [1]. We use these parameters as the following settings. The gain of the transmit antenna ($G_{t}$) and the gain of the receive antenna ($G_{r}$) are identical, 5000. The range from the radar to the target in meters, *R*, is 50,000–100,000 m. The instantaneous receiver bandwidth, *B*, is 30 MHz. *F* is the noise figure of the receiver subsystem and is set as one

An additional attenuation is introduced into Equation (8) in [1], and this attenuation is calculated with respect to Equation (2). The frequency of the plasma is 6π×10${}^{9}$ (the altitude is 100 km) to 14π×10${}^{9}$ (the altitude is 20 km) rad/s [15], with a linear prediction model. The collision frequency of the plasma is 10${}^{6}$ (the altitude is 100 km)–10${}^{7}$ (the altitude is 20 km) 1/s [15], also with a linear prediction model. The depth of the plasma is identically approximated as 5 m.

In addition, to know the applicability of the proposed performance evaluation indexes in blackout condition, the rain attenuation and the carrier’s high maneuvering characteristic have not been considered in these experiments.

### 4.2. Performance Evaluation Results for an X-Band Radar and the Analysis

**Figure 4:**
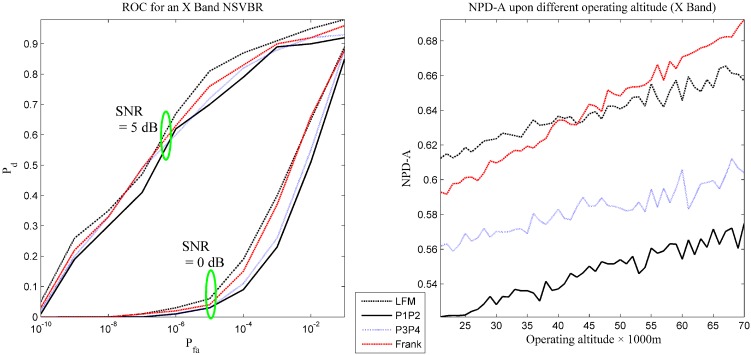
The receiver operating curve and the NPD-A of Scenario 1.

The performance evaluation results are presented in Table 1 and Figure 4. In Figure 4, to keep a clear interface, the results of P1 and P2 are averaged and combined into one curve (the performance of these two waveforms is similar). This is also true for P3 code and Frank code. In Table 1, Table 2 and Table 3, all of the results of NPD-A are the mean value when the altitude of the vehicle is between 21,000 m and 70,000 m. Because upper case subscripts may confuse the symbols, the subscripts of $P_{D}$ and $P_{FA}$ are lower case in the following figures.

According to Table 1 and Figure 4, the following essential information can be obtained.

(1) The detection performance for all of the tested waveforms is not ideal in this band.

(2) The performance of LFM and Frank code are a little better than other waveforms, while that of P1 code and P2 code is typically below the expected value.

(3) The proposed performance evaluation indexes can describe the detection performance in many aspects of interest.

**Table 1:** Results of the performance evaluation indexes. NPD, normalized probability of detection; M, mission; A, altitude; RNPD, robustness of NPD; T, time; LFM, linear frequency modulation.

Waveform	NPD-M	NPD-A	RNPD-T	RNPD-A
LFM	0.67	0.64	0.59	0.55
P1	0.56	0.56	0.48	0.50
P2	0.60	0.61	0.52	0.53
P3	0.62	0.61	0.53	0.54
P4	0.63	0.61	0.52	0.55
Frank	0.66	0.63	0.60	0.54

### 4.3. Performance Evaluation Results for a Ku-Band Radar and the Analysis

**Figure 5:**
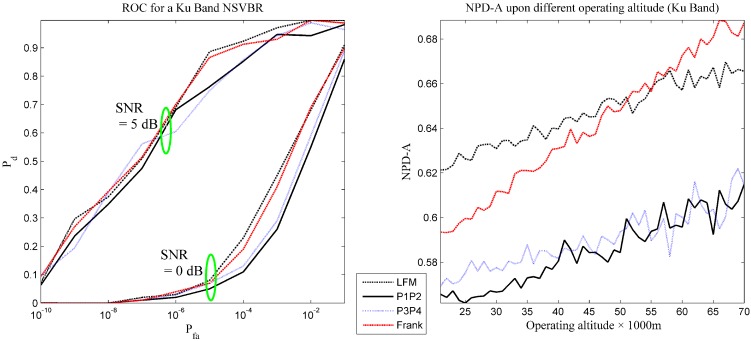
The receiver operating curve and the NPD-A of Scenario 2.

The performance evaluation results are presented in Table 2 and Figure 5. In Figure 5, the combination rule is identical to that in Figure 4.

With the data presented in Table 2 and Figure 5, the following information can be achieved.

(1) The detection performance for all of the tested waveforms is better than that in the X-band. This is in accordance with the anticipation that higher carrier frequency brings benefits for target detection in blackout condition [15].

(2) There is still a difference in the detection performance for different waveforms. Similar results hold for the tested waveforms: LFM and Frank code maintain better performance, while P1 code and P2 code have lower performance than other waveforms.

(3) For these waveforms, the curve of performance tends to be clustered in time, which means the performance difference is not significant. Even in this case, our performance indexes can figure out the difference.

**Table 2:** Results of the performance evaluation indexes.

Waveform	NPD-M	NPD-A	RNPD-T	RNPD-A
LFM	0.69	0.65	0.60	0.63
P1	0.63	0.62	0.55	0.54
P2	0.59	0.58	0.51	0.53
P3	0.64	0.60	0.58	0.56
P4	0.65	0.64	0.59	0.61
Frank	0.66	0.62	0.67	0.64

### 4.4. Performance Evaluation Results for a Ka-Band Radar and the Analysis

**Figure 6:**
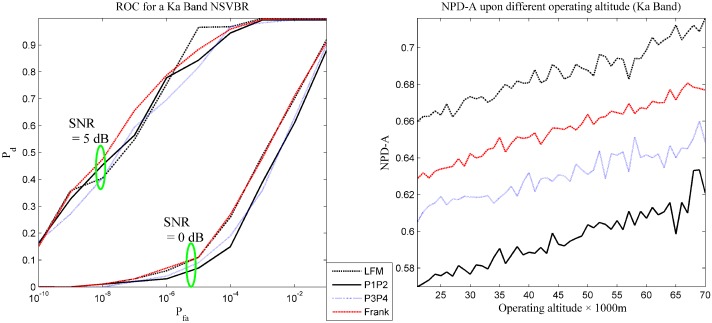
The receiver operating curve and the NPD-A of Scenario 3.

The performance evaluation results are presented in Table 3 and Figure 6. Similarly, in Figure 6, the combination rule is identical to that in Figure 4.

With the knowledge of the previous two experiments, some useful information can be acquired with respect to Table 3 and Figure 6.

**Table 3:** Results of the performance evaluation indexes.

Waveform	NPD-M	NPD-A	RNPD-T	RNPD-A
LFM	0.72	0.69	0.61	0.66
P1	0.59	0.60	0.52	0.56
P2	0.64	0.65	0.56	0.59
P3	0.64	0.67	0.62	0.65
P4	0.65	0.66	0.59	0.58
Frank	0.69	0.64	0.68	0.70

(1) The detection performance for all of the tested waveforms is better than that in the X-band, while having minor improvement than that in the Ku-band. The improvement in the Frank code is the greatest.

(2) The proposed performance evaluation indexes are still applicable in this scenario and can describe the detection performance in many fundamental aspects, like the operating condition considered, the robustness upon altitude calculated, *etc*.

(3) The variation in NPD-A can mostly be attributed to the change of blackout condition.

(4) As has been figured out by other scientists, the choice of pulse waveform affects the performance of target detection [1] and is confirmed by the experiments.

It should be noted that the curves in the simulation output are not as flat as expected; this is because the sampling interval of $P_{FA}$ is significantly longer than the expected interval. Working with the planning interval may achieve better images, while the experimental load increases.

### 4.5. Experiments of the Overall Performance upon Different Carrier Frequencies

**Figure 7:**
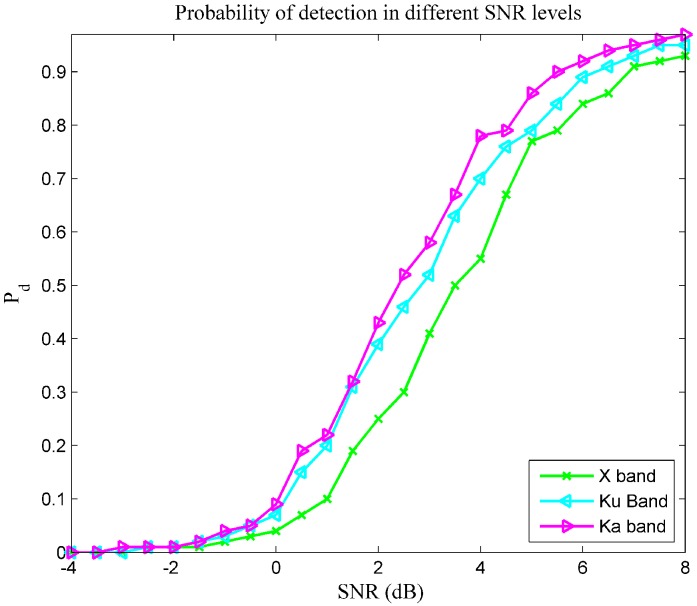
The detection performance as a function of SNR.

To make this point clearer, Figure 7 plots the detection performance as a function of SNR. The results of $P_{d}$ are arrived at from those detection processions with identical SNR in a given frequency, regardless of the waveform and operating altitude. There is a constant false alarm rate (CFAR) rule applied in this experiment, and $P_{FA}$ is 10${}^{-4}$. This figure shows that, for an NSVBR, a detector will maintain better performance when working with a Ka band EM wave, compared to the same detector when working with an X-band EM wave.

## 5. Discussion

### 5.1. Comparison between the Existing Methods and This Work

With respect to the above-mentioned simulation results, a comparison between the existing performance evaluation methods and the proposed measures is performed in Table 4.

**Table 4:** The comparison between the existing technologies and the proposed methodology. Legend: ★, high achievement; •, satisfactory; ▴, improvement needed; ■, unsatisfactory.

Aspect	$P_{D}$ & $P_{FA}$	ROC	New Measures
Q1	•	▴	★
Q11	■	■	•
Q2	★	•	★
Q3	■	•	•
Q4	•	■	•
Q5	•	•	•

The meanings of some symbols in Table 4 are listed below.

- Q1: Is the methodology applicable to the target detection performance evaluation of an NSVBR and flexible for a complicated scenario (such as, in blackout condition)?
- Q11: Is the methodology flexible for considering multiple performance aspects in blackout condition?
- Q2: The precision of the method.
- Q3: The generalization of the method.
- Q4: Is the method easy to configure?
- Q5: The computational load of the method.

According to the information in Table 4, our method offers a good choice for target detection performance evaluation of an NSVBR, especially in blackout condition. Further, the proposed evaluation indexes do not engage in the detecting process, and the evaluation output maintains good objectiveness. However, as can be identified in Table 4, our measures have more computational load than the conventional approaches, though affordable for a personal computer.

Since this work focus on developing performance evaluation methods for target detection of an NSVBR, the primary contributions are some performance indexes. The potential uncertainties and errors of them (in application) are mostly from the insufficient sample size. As shown in Table 1, Table 2, Table 3 and Table 4, if the sample size is large enough, the precision (accuracy) of these indexes can meet the system requirements.

### 5.2. Comparison between Radars with Different Installations

Given the experiments of an NSVBR, we make a comparison between spaceborne, near-space vehicle-borne and airborne radars, with reference to related materials [1,2,26]. The information regarding the basic characteristics is presented in Table 5. It is clear that the NSVBR is an essential supplementary for the existing radar installations.

**Table 5:** The comparison between radars with different installations. Coverage is largely dependent on altitude and accessible incidence angle.

Aspect	Spaceborne Radar	NSVBR	Airborne Radar
Basic Functions	SAR imaging	Target search/detect/track, SAR imaging	Target search/detect/track, SAR imaging
Range of observation	Some 100 km	Some 50 km	Some 10 km
Revisiting time	Some 10 h	Mostly less than 1 h	Mostly some hours
Coverage	Some 10,000 km${}^{2}$	Some 2500 km${}^{2}$	Some 100 km${}^{2}$
Resolution (SAR)	<1 m to some 10 m	<1 m	<1 m to some 10 m
Overfly time	Imposed by orbit	Adjustable	Adjustable
Maneuvering ability	Poor	Highly	Moderate

## 6. Conclusions

In this paper, performance evaluation measures are investigated for target detection of a near-space vehicle-borne radar in blackout condition. The advantages of our evaluation indexes are that they can provide detailed information about the detection process, and some of these evaluation indexes can be employed in the performance evaluation of NSVBR detection in other conditions. Given these contributions, though, the newly-proposed approach involves more complicated computations than the conventional index, the probability of detection. Future work on this topic is validating the evaluation indexes with extensive field test data.
